# Effects of Changes in Background Colour on the Identification of Own- and Other-Race Faces

**DOI:** 10.1177/2041669519843539

**Published:** 2019-04-13

**Authors:** Catriona Havard, Stephanie Richter, Martin Thirkettle

**Affiliations:** School of Psychology, The Open University, UK; Department of Psychology, Sociology and Politics, Sheffield Hallam University, UK

**Keywords:** colour, face perception, memory, perception, search/exploration, social cognition, visual memory

## Abstract

The current study investigated whether small differences in the background colours between the lineup members would influence identification accuracy of own-race and other-race faces. Using the well-established 1-in-10 paradigm, half of the array faces had exactly the same backgrounds, and half were on backgrounds of slightly different hues of green. For target present arrays, participants were more accurate at identifying own-race faces when compared with the other-race faces when all backgrounds were the same. However, when backgrounds had slightly different hues, there was no difference in how accurate people were at identifying faces from both races. For target absent arrays, participants were more likely to incorrectly choose a face if the backgrounds were not all the same, regardless of the race of faces. Real-world implications from these findings are that using lineups where the backgrounds are slightly different hues may increase the likelihood of the false identification of innocent suspects.

## Introduction

Identification parades are one of the most common means of identifying a perpetrator of a crime and can be powerful evidence in securing convictions in criminal cases. In an identification parade (also known as a lineup), a suspect is placed among a number of similar looking people (foils), and the witness is asked to either select the person they recognise as being the culprit or state the culprit is not there. However, eyewitness evidence is notoriously error prone, with organisations such as the Innocent Project finding that 75% of wrongful convictions that were later exonerated had verdicts based on faulty eyewitness evidence, resulting in mistaken identity (https://www.innocenceproject.org/).

One way to help reduce mistaken identity is to eliminate any bias in the lineup. Considerable eyewitness research has investigated how to ensure lineups are not biased and to reduce false identifications of innocent suspects. Investigations have included the use of unbiased instructions ‘*the person may or may not be there*’ (Malpass & Devine, 1981; Steblay, 1997), the sequential presentation of lineups, presenting each lineup member individually rather than simultaneously (Lindsay et al., 1991; Steblay, Dysart, Fulero, & Lindsay, 2001; Wells, Steblay, & Dysart, 2015) and double blind administration, where the administer does know not the identity of the suspect (Greathouse & Kovera, 2009; Phillips, McAuliff, Kovera, & Cutler, 1999). However, fewer studies have investigated whether inconsistencies in the images used in the lineup can influence identification accuracy.

Buckhout, Figueroa, and Hoff (1975) used biased instructions and also a biased photo array (2 × 3) where the target’s photo was at a different angle to the rest of the photos, and they had a different facial expression. In the biased lineup with biased instructions, witnesses were 20% more likely to identify the target when compared with the unbiased lineups. In a subsequent mock witness task, where witnesses were shown the lineup, but not the preceding mock crime, the findings still revealed that the target was chosen above chance in the biased lineup. Unfortunately, this study only used target present (TP) lineups, and so it was not able to ascertain whether the biased lineups would also influence false identifications of innocent suspects.

The aim of the current study was to determine whether small differences in the background colours between the lineup members would influence identification accuracy. There are currently two types of identification software used in the United Kingdom; video identification procedure electronic recording (VIPER) and profile matching (PROMAT). Although both systems have a standard background, variations in lighting and cameras have resulted in differences in the hues of background for lineup members. In VIPER lineups, individuals are placed on a grey background with standardised lighting, and each recording is sent to the VIPER headquarters for checking before being approved, resulting in fewer differences (for details, please see Havard, Memon, Clifford, & Gabbert, 2010; VIPER®, 2018). In contrast, PROMAT places individuals on a green background that can be filmed either in a custom booth or in front of a green screen (for details, please see PROMAT Digital Image Capture Booth, 2010). As a result, PROMAT has the greatest variations in background colour, which possibly result from the use of different lighting across photographs and no formal checking procedures (PROMAT, 2010). Such variations in hue, while fairly small in chromatic value, are almost certainly perceptible by observers, not least because the human eye is able to discriminate more hues of green, when compared with other colours (Mullen & Kulikowski, 1990). Currently, no research has investigated whether the differences in background hues influence identification accuracy and whether this would further influence the recognition of targets that are of the same or a different race to the viewer.

### Other-Race Effect

A fairly robust effect in the literature is the finding that people are generally better at recognising faces from their own race when compared with other races. This bias has been referred to as the other-race effect, own-race bias, own-group bias, and cross-race effect. The own-race bias (ORB; the term we will use here) appears to be greater in Caucasian Europeans when compared with other racial groups (Hancock & Rhodes, 2008; Jackiw, Arbuthnott, Pfeifer, Marcon, & Meissner, 2008; Walker & Hewstone, 2006). The ORB has been confirmed by meta-analyses of face recognition studies that have revealed that participants are more likely to correctly recognise previously seen own-race faces and more likely to falsely recognise other-race faces that have not been seen previously (Bothwell, Brigham, & Malpass, 1989; Meissner & Brigham, 2001).

The ORB has also been replicated using the eyewitness paradigm, where a witness views a mock crime and then a lineup or photo array. A number of studies have found that in the lineup task, witnesses are much more likely to correctly identify someone who is the same race, and they are more likely to misidentify or falsely identify an innocent who is of a different race (Havard, Memon, & Humphries, 2017; Jackiw et al., 2008; Kask & Bull, 2009; Smith, Stinson, & Prosser, 2004). This supports findings from the Innocent Project which has found that of those who were convicted of mistaken identification, nearly half (49%) involved cross-race identification (Garrett, 2011)

An alternative, well-established paradigm is the 1-in-10 face recognition task (Bruce et al., 1999; Henderson, Bruce, & Burton, 2001; Megreya & Burton, 2006) that has the advantage, unlike the typical eyewitness task, of being able to use multiple trials. This paradigm has also replicated the ORB, where both U.K. and Egyptian participants were more accurate with own-race faces when compared with the other-race faces. U.K. participants in particular were much more likely to misidentify or make a false positive (FP) response to Egyptian faces (Megreya, White, & Burton, 2011). The false identification of faces has some real-world implications, especially if those who are misidentified are wrongfully convicted of crimes they did not commit. If the image properties of lineups are in anyway partly responsible for any wrongful convictions, due to the suspect standing out from the other lineup members, then this is a noteworthy area of research.

## The Current Study

In the current study, we predict that using photo arrays with variety of green hues for the backgrounds will influence identification accuracy using a 1-in-10 paradigm. For TP lineups, using the mixed backgrounds will either increase the correct identifications as the target stands out from the other faces, or it may reduce accuracy, as other faces may appear more salient when compared with the target face. For TA lineups, there may be more false identifications for faces on the mixed backgrounds, as some faces may stand out, and this may encourage participants to inaccurately choose when compared with photo arrays with uniform backgrounds. We also predict the data will show an ORB where participants will be more accurate at identifying faces that are the same race when compared with faces of another race. There may also be an interaction between background variability and the identification of own- and other-race faces.

## Methodology

### Participants

A total of 22 participants took part in the study (14 female, ages 23–63, *M* = 41.5). All were Caucasian and were members of staff from the Open University. They were reimbursed with a shopping voucher for their time.

### Stimuli and Procedure

The PROMAT software has a database of more than 26 thousand images, so to refine the sample to be used for the background colour manipulation, search terms were created to select a sample that would be representative of the average population of suspects. Using PROMAT (2010) to build a lineup, searches were conducted with the following preestablished criteria: age (minimum 18 years, maximum 35 years), build (medium, no glasses), hair (black), and for ethnicity “White” was selected. The first 100 faces were chosen and saved, as unless an officer asks for the faces to be randomised, these are the faces that will appear initially when creating a lineup. The same search terms were used again except removing the White ethnicity and replacing it with Asian, Oriental, and Black (these are the software and not authors’ terms). For each different ethnicity, the first 100 faces were used, apart from for the Oriental search as there were only 95 returns. This resulted in a total of 395 faces on various green backgrounds.

**Figure 1.**
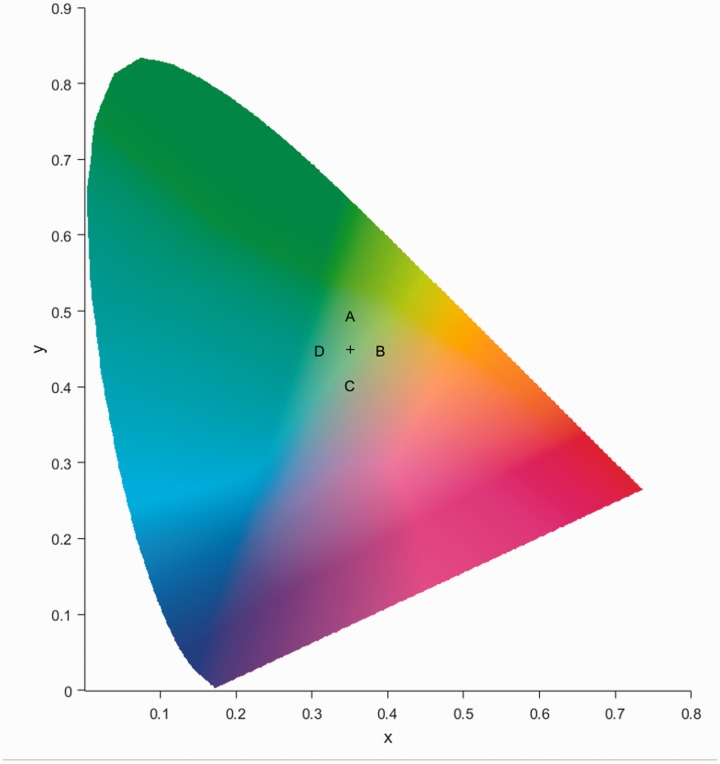
Variability of colour values in CIE 1931 *xy* coordinates. The mean for the set of 395 PROMAT images is marked by the “+” sign; each of the four letters mark one of the experimental background colour values of 2 standard deviations from the mean value along each axis of CIE space.

The 395 faces had quite a bit of variability between the green backgrounds. There are two reasons for this. Many of the first filler or foil lineup members were filmed in a custom-built booth with a green background and uniform lighting. However, as these booths can be costly, subsequent fillers and suspects are often filmed at a police station without a booth and in front of a much less expensive green screen. If a suspect is filmed in front of a green screen, the lighting in the police station will have a great influence on how the background appears. To determine the variability between the faces and to create a mean background value that could be experimentally manipulated, MATLAB software was used. The mean background colour was determined for the 395 faces after segmenting the face from the background. The pixel values from these backgrounds were then converted from the RGB values of the image files into CIE 1931 XYZ coordinates, a commonly used colour space, to allow the colour value of the background, the chromaticity, to be assessed and manipulated independently from luminance information (please see Figure 1). Once the mean chromaticity value for all faces had been found, manipulated values of 2 standard deviations (SD) away from the mean in both the X and Y axes in CIE 1931 XYZ space were calculated (see Table S1 in Supplementary Materials for CIEXYZ and CIELab values for background colours).

These backgrounds were then used to make experimental versions of the original face images for testing. Initial pilot testing used manipulated backgrounds that were 1 SD away from the mean; however, participants could not reliably distinguish from the mean background; therefore, values of 2 SD away from the mean chromatic values of the 395 images used were used to ensure comparability between the experimental stimuli and the original data set of PROMAT images.

**Figure 2.**
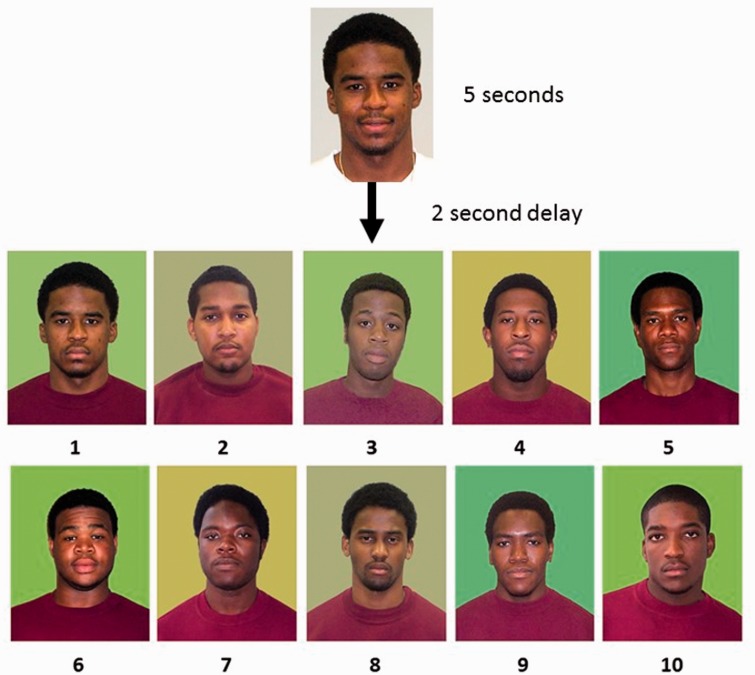
Each trial begins with the target face for 5 seconds, and after a delay of 2 seconds, an array of 10 faces was presented. A target present trial on mixed background is shown, and the correct answer here is No. 1.

The 1-in-10 face recognition paradigm was employed. The experiment was presented using E-Prime software on an Alienware computer, and responses were recorded with a keyboard. The monitor was 21″, and participants had a viewing distance of 50 cm. Prior to the experiment, the participants were given instructions. Each trial began with the presentation of an image of a colour face (approximately 8 × 12 cm), presented for 5 seconds, on a grey background. Then, after a delay of 2 seconds, an array of 10 faces (2 rows of 5) appeared, where each face was presented on a green square and all were presented on a white background. Each array face was approximately 3.5 × 5 cm in size. Each array face was numbered (please see Figure 2), from 1 through 10, and they were displayed until the participants responded. The images were taken from a database that had been used in previous research by Meissner, Brigham, and Butz (2005).

Participants were tested individually in a session that lasted approximately 30 minutes, where they were presented with 160 trials. Of these, 80 trials had Caucasian targets and arrays, and 80 were African American targets and arrays. Half the arrays were TP, where the target face was present in the array, and half were target absent (TA) arrays, where the target face was not present in the array. When the target was present in the array, a different target image was used. For half of the arrays, all the squares were the mean colour, and for the remaining half of the arrays, the backgrounds consisted of the four colour variations, 2 SD from the mean, as well as the mean background. Therefore, there were five different coloured backgrounds that were randomly assigned. The arrays were counterbalanced so that the target appeared in every location in the array.

## Results

Table 1 shows the mean performance of recognition accuracy for faces in both the mean (all mean coloured squares) and mixed (all four colours 2 SD from the mean along with the mean coloured background), for Caucasian and African American arrays. For TP arrays, three measures were calculated: hits (correctly identifying the target), misses (incorrectly saying the target was not present) and misidentifications (MisID: incorrectly choosing someone other than the target). For TA arrays, two measures were calculated: correct rejections (CR: correctly stating the target is not there) and FPs (choosing someone from the array).

**Table 1.** Percentage of Accuracy and of Hits, Misses, and Misidentifications for Caucasian and Afro-American Targets on Mean and Mixed Arrays (Standard Deviations in Parentheses).

Array condition	Accuracy	Hit	Miss	MisID	FPs
Caucasian Mean array	69.43 (10.80)	71.59 (15.54)	16.36 (14.07)	12.05 (8.26)	32.73 (17.84)
Caucasian Mixed array	66.48 (13.58)	67.27 (16.81)	16.14 (13.62)	16.59 (12.19)	34.32 (19.51)
African American Mean array	55.91 (13.62)	56.36 (18.01)	18.18 (10.07)	25.45 (12.90)	44.55 (19.51)
African American Mixed array	55.34 (13.39)	62.95 (16.52)	13.64 (9.28)	23.41 (13.22)	52.27 (18.94)

*Note*. FPs = false positives.

### Overall Accuracy

Overall accuracy data were subjected to a repeated measures analysis of variance with race (Caucasian vs. African American) and array background (mean vs. mixed). There was a main effect of race, *F*(1, 21) = 34.61*, p* < .001, $ɳ{\text{p}}^{2}$ = .62, but no reliable main effects of array background, *F*(1, 21) = 1.153, *p* = .29, ɳp2 = .05, nor significant interaction for race and array background, *F*(1, 21) = 0.58, *p* = .29 ɳp2 = .03. The main effect of Race revealed that participants were significantly more accurate with Caucasian faces when compared with African American faces (67.96 vs. 55.63).

### Array Responses

Separate repeated measures analyses of variance were conducted on the different types of responses that could be given; hits (correctly identifying the target face), misses (incorrectly saying the target is not there) and MisID (identifying someone other than the target) for the TP lineups and FPs (incorrectly identifying a face) for the TA lineups. For hits, there was a significant interaction for race and background, *F*(1, 21) = 5.83, *p* = .025, ɳp2 = .22, and a main effect for race, *F*(1, 21) = 12.57, *p* = .002, ɳp2 = .37. Follow-up analyses found that there were more hits for the Caucasian targets when compared with African American targets for the mean backgrounds (*p* < .001), but no significant differences were found in the hits for both races of target faces on mixed backgrounds (*p* = .23). For Caucasian faces, there were slightly more hits for the mean when compared with the mixed background arrays; however, this was not statistically significant (*p = *.17). In contrast, for the African American faces, there were more hits for the mixed arrays when compared with the mean arrays; however, this did not reach statistical significance (*p* = .057). For the misses, there were no significant main effects or interactions (all *p*s > .1). For MisIDs, there was a significant effect of race, *F*(1, 21) = 18.49, *p* < .001, ɳp2 = .47, but no effect for array, *F*(1, 21) = .304, *p* = .30, ɳp2 = .01, and no significant interaction for race and array, *F*(1, 21) = 3.67, *p* = .07, ɳp2 = .15. There were significantly more MisIDs for African American targets when compared with Caucasian targets.

For the FPs from the TA arrays, there was a significant effect for race, *F*(1, 21) = 27.53, *p* < .001, ɳp2 = .57, and a significant effect of background, *F*(1, 21) = 7.21,* p* = .014, ɳp2 = .26, but no significant interaction, *F*(1, 21) = 2.58, *p* = .123, ɳp2 = .11. There were significantly fewer FPs for Caucasian arrays (*M* = 33.52, *SE* = 3.73) when compared with African American arrays (*M* = 48.41, *SE* = 3.92). There were also significantly fewer FPs for the mean backgrounds (*M* = 38.64, *SE* = 3.58) when compared with the mixed background (*M* = 43.30, *SE* = 3.74).

## Discussion

The aim of the study was to investigate whether variability in the hues of the background of photo arrays would affect the accurate identification of target faces. We report that, while the presence of variation in background colour seems not to affect identification performance when a target is present, FPs are reduced in TA arrays when background colour is held constant; that is, witnesses are more likely to incorrectly identify an innocent face from a lineup when background variations, no greater than those found in commercial lineup databases, are present. Previous research has shown that biased lineups can improve the correct identification of targets for TP lineups by making the target stand out from the other lineup members (Buckhout et al., 1975). This was not the case for the current study, where the mixed backgrounds did not increase target identification. This could result from methodological differences; in previous research, the target differed from the rest of the foils in the biased lineup, while in the current research, the background of the target and the foils all varied from one another in the mixed condition. Previous research has found that biased lineups can increase false identifications for TA lineups, when it appears the witness must choose someone from a lineup (Malpass & Devine, 1981; Steblay, 1997). This was also a finding of the current study, where FPs were increased for the mixed backgrounds; it could be that some faces appeared more salient, encouraging participants to choose a foil.

A further aim was to investigate whether the background variability would have a greater influence on identifying own, or other-race faces, and thereby increase or decrease the ORB. We predicted participants would be more accurate with own-race (Caucasian) faces when compared with other-race (African American) faces. This prediction was supported as overall accuracy was greater for own-race faces, when compared with other-race faces, and replicated previous research (Bothwell et al., 1989; Hancock & Rhodes, 2008; Havard et al., 2017; Jackiw et al., 2008; Megreya et al., 2011; Meissner & Brigham, 2001; Walker & Hewstone, 2006). Interestingly, the accuracy data revealed that even with own-race faces, participants were still incorrect nearly a third of the time, which replicates other studies using this paradigm that report face matching to be highly error prone (Megreya & Burton, 2006, 2007, 2008; Megreya et al., 2011). Although, the accuracy data revealed an ORB, when responses for TP and TA arrays with mean or variable backgrounds were taken into account, the relationship appeared to be more complex.

For TP arrays that contained mean backgrounds, there were significantly more hits for the own-race faces when compared with the other-race faces. This replicated the ORB described in numerous other studies that have reported higher accuracy for identifying own-race faces (Hancock & Rhodes, 2008; Jackiw et al., 2008; Walker & Hewstone, 2006). In contrast, for the mixed arrays, the hit rates for both the own- and other-race faces were not statistically different from one another, and thereby, the ORB was reduced. The variations in background colours seemed to slightly reduce the accuracy for identifying Caucasian (own-race) faces and slightly increase the accuracy for identifying African American (other-race) faces. However, it should be noted that using the mixed arrays did not significantly affect the hit rate for either the own- and other-race faces when compared with the mean background arrays. So we should be cautious in making any assumptions about it reducing performance for the own-race faces and enhancing accuracy for other-race faces.

When it came to choosing someone other than the target from the TP array, there were fewer mistaken identifications for the own-race faces when compared with other-race faces. This ORB bias of mistaken identity was found for both the mean and mixed arrays and replicates previous research (Havard et al., 2017; Jackiw et al., 2008; Kask & Bull, 2009; Smith et al., 2004). The background array variations did not significantly influence the misidentification rate, which suggests other lineups members did not appear to be more salient than the targets, when there were variations in the backgrounds.

Background variations in the array, and also the race of the target, did not seem to influence whether participants incorrectly said the target was not there, that is, a miss response. Previous research using this paradigm found that U.K. participants were more likely to make a miss response for own-race (Caucasian) faces when compared with other-race (Egyptian) faces (Megreya et al., 2011). The previous study not only used a different stimuli set but had the added condition of inverting half of the stimuli, and the miss rates varied considerably more than the current study. In the current study, the miss rates were relatively low and similar across conditions.

When it came to falsely choosing from TA arrays, as predicted, there were fewer FPs for own-race faces when compared with other-race faces, confirming the ORB. This finding replicated previous research and meta-analyses reporting higher FP identifications of individuals who are of a different race (Havard et al., 2017; Jackiw et al., 2008; Kask & Bull, 2009; Meissner & Brigham, 2001; Smith et al., 2004). Interestingly, using the mean backgrounds increased the accuracy for both the own- and other-race faces, by reducing FPs. This suggests that using uniform backgrounds for arrays has the potential to reduce bias and false identifications of innocent suspects for both same race and other-race faces. Alternatively, when there are variations in the background hues, this could potentially increase false identifications of innocent suspects.

## Future Research and Conclusions

The current study uses the well-established 1-in-10 paradigm to include multiple trials and to replicate previous research on the ORB bias. To ensure generalisability of findings, future research could include examining the variability of backgrounds using the eyewitness paradigm. Furthermore, future research could use different samples with different ethnicities to investigate whether the ORB and the use of different backgrounds generalises to different populations. Alternatively, future research could potentially investigate the effect different, uniform backgrounds have on identification performance (if any) across races in order to provide an evidence base for choice of background colour in lineup images. Lastly, future research should investigate the relationship between variation in the background colour of lineup images, ethnicity of the face and identification performance. Different ethnic groups have dramatically different face colours, and it would be potentially very useful to quantify the impact differences in background colour have on lineup images of different ethnicity faces.

The findings from this study have real-world implications for reducing cases of mistaken identification that could lead to wrongful convictions. Although there are some factors, such as the ORB, which cannot be controlled by the police and increase the chance an innocent suspect is falsely identified. Other factors such as having uniform lineup backgrounds can be controlled to ensure that any lineup member does not stand out and to reduce false identifications that can lead to wrongful convictions.

## Supplemental Material

Supplemental material for Effects of Changes in Background Colour on the Identification of Own- and Other-Race Faces Click here for additional data file. Supplemental Material for Effects of Changes in Background Colour on the Identification of Own- and Other-Race Faces by Catriona Havard, Stephanie Richter and Martin Thirkettle in i-Perception

## References

[bibr1-2041669519843539] Bothwell R. K. Brigham J. C. Malpass R. S. (1989) Cross-racial identifications. Personality and Social Psychology Bulletin 15: 19–25. doi:10.1177/0146167292183005.

[bibr2-2041669519843539] Bruce V. Henderson Z. Greenwood K. Hancock P. J. B. Burton A. M. Miller P. (1999) Verification of face identities from images captured on video. Journal of Experimental Psychology: Applied 5: 339–360.

[bibr3-2041669519843539] Buckhout R. Figueroa D. Hoff E. (1975) Eyewitness identification: Effects of suggestion and bias in identification from photographs. Bulletin of the Psychonomic Society 6: 71–74.

[bibr4-2041669519843539] Garrett B. L. (2011) Convicting the innocent: Where criminal prosecutions go wrong, Cambridge, MA: Harvard University Press.

[bibr5-2041669519843539] Greathouse S. M. Kovera M. B. (2009) Instruction bias and lineup presentation moderate the effects of administrator knowledge on eyewitness identification. Law and Human Behavior 33: 70–82. 18594956 10.1007/s10979-008-9136-x

[bibr6-2041669519843539] Hancock K. J. Rhodes G. (2008) Contact, configural coding and the other-race effect in face recognition. British Journal of Psychology 99: 45–56. doi:10.1348/000712607X199981. 17535471 10.1348/000712607X199981

[bibr7-2041669519843539] Havard C. Memon A. Clifford B. Gabbert F. (2010) A comparison of video and static photo lineups with child and adolescent witnesses. Applied Cognitive Psychology 24: 1209–1221.

[bibr8-2041669519843539] Havard C. Memon A. Humphries J. E. (2017) The own-race bias in child and adolescent witnesses: Evidence from video lineups. International Journal of Police Science & Management 19(4): 261–272.

[bibr9-2041669519843539] Henderson Z. Bruce V. Burton A. M. (2001) Matching the faces of robbers captured on video. Applied Cognitive Psychology 15: 445–464.

[bibr10-2041669519843539] Jackiw L. B. Arbuthnott K. D. Pfeifer J. E. Marcon J. L. Meissner C. A. (2008) Examining the cross-race effect in lineup identification using Caucasian and First Nations samples. Canadian Journal of Behavioural Science 40: 52–57.

[bibr11-2041669519843539] Kask K. Bull R. (2009) The effect of different presentation methods on multi-ethnicity face recognition. Psychology, Crime & Law 15: 73–89.

[bibr12-2041669519843539] Lindsay R. C. L. Lea J. A. Nosworthy G. J. Fulford J. A. Hector J. LeVan V. Seabrook C. (1991) Biased lineups: Sequential presentation reduces the problem. Journal of Applied Psychology 76: 796–802. 1774217 10.1037/0021-9010.76.6.796

[bibr13-2041669519843539] Malpass R. S. Devine P. G. (1981) Eyewitness identification: Lineup instructions and the absence of the offender. Journal of Applied Psychology 66: 482–489.

[bibr14-2041669519843539] Megreya A. M. Burton A. M. (2006) Unfamiliar faces aren’t faces: Evidence from a matching task. Memory & Cognition 34: 865–876. 17063917 10.3758/bf03193433

[bibr15-2041669519843539] Megreya A. M. Burton A. M. (2007) Hits and false positives in face matching: A familiarity based dissociation. Perception & Psychophysics 69: 1175–1184. 18038955 10.3758/bf03193954

[bibr16-2041669519843539] Megreya A. M. Burton A. M. (2008) Matching faces to photographs: Poor performance on eyewitness memory (without the memory). Journal of Experimental Psychology: Applied 14: 364–372. 19102619 10.1037/a0013464

[bibr17-2041669519843539] Megreya A. M. White D. Burton A. M. (2011) The other-race effect does not rely on memory: Evidence from a matching task. Quarterly Journal of Experimental Psychology 64: 1473–1483. 10.1080/17470218.2011.575228 21812594

[bibr18-2041669519843539] Meissner C. A. Brigham J. C. (2001) Thirty years of investigating the own-race bias in memory for faces: A meta-analytic review. Psychology, Public Policy, and Law 7: 3–35. doi:10.1037// 1076-89/1.7.1.3.

[bibr19-2041669519843539] Meissner C. A. Brigham J. C. Butz D. A. (2005) Memory for own and other-race faces: A dual-process approach. Applied Cognitive Psychology 19: 545–567. doi:10.1002/acp.1097.

[bibr20-2041669519843539] Mullen K. T. Kulikowski J. J. (1990) Wavelength discrimination at detection threshold. Journal of the Optical Society of America 7: 733–742. 2338595 10.1364/josaa.7.000733

[bibr21-2041669519843539] Phillips M. R. McAuliff B. D. Kovera M. B. Cutler B. L. (1999) Double-blind photoarray administration as a safeguard against investigator bias. Journal of Applied Psychology 84: 940–951.

[bibr22-2041669519843539] PROMAT. (2010). Promat video ID system: Image capture [Computer software]. Lancashire, England: Promat Envision International.

[bibr23-2041669519843539] PROMAT Digital Image Capture Booth. (2010). Retrieved from http://www.promatenvision.co.uk/promat.aspx?section=products&product=booth.

[bibr24-2041669519843539] Smith S. M. Stinson V. Prosser M. A. (2004) Do they all look alike? An exploration of decision-making strategies in cross-race facial identifications. Canadian Journal of Behavioural Science 36: 144–153.

[bibr25-2041669519843539] Steblay N. Dysart J. Fulero S. Lindsay R. C. L. (2001) Eyewitness accuracy rates in sequential and simultaneous lineup presentations: A meta-analytic comparison. Law and Human Behavior 25: 459–473. 11688368 10.1023/a:1012888715007

[bibr26-2041669519843539] Steblay N. M. (1997) Social influence in eyewitness recall: A meta-analytic review of lineup instruction effects. Law and Human Behavior 21: 283.

[bibr27-2041669519843539] VIPER®. (2018). *Video Identification Parade*. Retrieved from http://www.viper.police.uk/pages/commercial.html.

[bibr28-2041669519843539] Walker P. M. Hewstone M. (2006) A perceptual discrimination investigation of the own-race effect and intergroup experience. Applied Cognitive Psychology 20: 461–475. doi:1 0.1002/acp.1191.

[bibr29-2041669519843539] Wells G. L. Steblay N. K. Dysart J. E. (2015) Double-blind photo lineups using actual eyewitnesses: An experimental test of a sequential versus simultaneous lineup procedure. Law and Human Behavior 39: 1–14. 24933175 10.1037/lhb0000096

