# Supplementary material for: Effects of Changes in Background Colour on the Identification of Own- and Other-Race Faces
Source: Iperception. 2019 Apr 13;10(2):2041669519843539. doi: 10.1177/2041669519843539 (PMC6463333; doi:10.1177/2041669519843539)
Supplement: Supplemental material for Effects of Changes in Background Colour on the Identification of Own- and Other-Race Faces [file Supplemental_Material.pdf]

The effects of changes in background colour on the identification of own and other-race faces.

Catriona Havard, Martin Thirkettle, Stephanie Richter

Supplemental materials

Table S1. – CIEXYZ and CIELab values for the mean background colour of the PROMAT line up images, and the calculated background colours A, B, C and D

	CIEXYZ			CIE Lab		
Colour	X	Y	Z	L	a	b
Mean	58.7517	75.37	33.7402	89.566	-29.105	46.6531
Background A	65.3849	75.47	27.2297	89.6127	-13.845	56.0688
Background B	65.371	75.27	46.1332	89.5193	-13.474	31.6952
Background C	53.2778	75.35	23.5944	89.5567	-42.726	61.8503
Background D	52.051	75.39	40.4655	89.5754	-45.995	38.2149
